# The Impact of Additional Exercise Interventions on Physical Performance and Muscle Strength of Frail Patients After Open-Heart Surgery: A Randomized Trial

**DOI:** 10.3390/medicina61101812

**Published:** 2025-10-09

**Authors:** Vitalija Stonkuvienė, Raimondas Kubilius, Eglė Lendraitienė

**Affiliations:** Department of Rehabilitation, Faculty of Nursing, Medical Academy, Lithuanian University of Health Sciences, 44307 Kaunas, Lithuania; raimondas.kubilius@lsmu.lt (R.K.); egle.lendraitiene@lsmu.lt (E.L.)

**Keywords:** cardiac rehabilitation, open-heart surgery, additional interventions, physical performance, functional capacity, muscle strength

## Abstract

*Background and Objectives*: Frail patients after open-heart surgery often experience worse treatment outcomes in improving physical performance and muscle strength. As the functional recovery of frail patients after open-heart surgery is slower, conventional rehabilitation is frequently insufficient to achieve treatment goals. Therefore, the inclusion of additional exercise interventions in cardiac rehabilitation is becoming more relevant. The aim of this study was to assess and compare the effectiveness of additional exercise interventions—multicomponent and computer-based programs—applied along with conventional cardiac rehabilitation in improving the functional capacity and strength of frail patients after open-heart surgery. *Materials and Methods*: The population of this single-center, three-arm, parallel-group, randomized controlled trial comprised 153 frail patients aged more than 65 years who underwent open-heart surgery. All patients were randomized into three groups: control (CG, *n* = 51), intervention 1 (IG-1, *n* = 51), and intervention 2 (IG-2, *n* = 51). All groups received conventional rehabilitation program six times/week, while the IG-1 additionally received the multicomponent dynamic training program 3 times/week, and the IG-2, the combined computer-based program 3 times/week. The primary outcome measure was change in the Short Physical Performance Battery (SPPB) score. Secondary outcome measures included the 6 min walk distance (6MWD), peak workload, grip strength, and leg press. Primary and secondary outcome measures were assessed before and after cardiac rehabilitation. *Results*: A total of 138 patients completed rehabilitation (46 in each group), and their data were included in the main analysis that followed a per-protocol approach. Although significant differences in the primary outcome—the SPPB score—were found in each group while performing within-group comparisons (*p* < 0.001), no significant pre-to-post rehabilitation differences were observed compared to all three groups (*p* = 0.939), and the effect sizes were small. Regarding secondary outcome measures, within-group comparison revealed significant differences in all parameters of all groups (*p* < 0.05), except for the grip strength of both hands in the IG-1. Between-group comparisons showed that the pre-to-post 6MWD difference between the CG and the IG-1 was significant (*p* = 0.014), but the effect size was small (ES = 0.240). Moreover, significant pre-and-post leg press 1RM differences (*p* < 0.001) were found between the CG and the IG-1 as well as between the CG and the IG-2 with the effect sizes being moderate (ES = 0.480) and large (ES = 0.613), respectively. *Conclusions*: Within-group comparison showed that all three rehabilitation programs are effective in improving almost all parameters of physical performance and muscle strength in frail patients after open-heart surgery. However, between-group comparisons indicated that computer-based interventions were more effective in improving leg press 1RM with a large effect size, while multicomponent training resulted in more effective gains in the 6MWD, although with a small effect size. These findings suggest that in clinical practice, computer-based exercise programs may be more suitable for patients with muscle weakness, while multicomponent exercise programs may be for those with reduced endurance.

## 1. Introduction

Based on the World Health Organization data, population aging is accelerating (WHO, 2024) [1]. On 1 January 2024, the total population of the European Union was 449.3 million, and more than one-fifth (21.6%) of it was older than 65 years [2]. It is estimated that the proportion of inhabitants aged more than 65 years will exceed 30% by the end of the 21st century [3]. It will have considerable implications for health care systems and national economies [4]. The prevalence of frailty is greater among older people with cardiovascular disease (CVD) than in the general population, and frail patients with CVD undergo open-heart surgery more frequently [5,6]. Patients with frailty are characterized by decreased physiological reserve and greater susceptibility to unfavorable health factors [7]. Frailty syndrome affects up to 30% of patients with coronary artery disease; 80% with heart failure; and 74% with aortic valve disease [5]. Studies show that patients with frailty are at a higher risk of postoperative complications, prolonged length of hospital stay, and functional decline [7,8].

Studies show that cardiac rehabilitation that involves physical activity programs can improve patients’ survival, reduce hospitalization and the likelihood of myocardial infarction, and improve quality of life [9]. The guidelines by the European Society of Cardiology highlight that cardiac rehabilitation must be comprehensive, multicomponent, individually tailored, and include physical activity promotion and integrated psycho-social support [10]. Such rehabilitation programs improve patients’ functional independence and help reduce the risk of CVD [11]. It has been reported that exercise-based cardiac rehabilitation can reduce all-cause mortality by 20% in patients with CVD [12]. Scientific literature mostly refers to multicomponent training [13]; however, other authors state that exercise interventions allowing to achieve better results during a shorter time should be considered [14]. For example, computer-based exercise programs provide feedback in real time that allows for assessing the result immediately after training [15,16], to adjust the session difficulty and increase exercise dose to individual capabilities [17,18], to monitor progress in real time to improve physical performance [19], and to tailor training individually [20].

Multicomponent exercise programs include several components: aerobics, strength, balance, and range of motion training. Scientific research shows that these programs are effective in patients with frailty and increase muscle strength as well as improve muscle endurance and balance [21]. Other programs, such as computer-based programs, are gaining more attention in modern cardiac rehabilitation [22]. It has been reported that interactive computer-based programs applied in rehabilitation are safe and effective in patients with frailty. These programs are fostering engagement in physical activity and enhancing it as well as allowing for individualization of a patient’s rehabilitation program and to correct it when needed [23]. There are ongoing discussions in the scientific community about which exercise programs are most effective in frail patients after a cardiac intervention [24,25].

The aim of this study was to assess and compare the effectiveness of additional exercise interventions—multicomponent and computer-based programs—applied along with conventional cardiac rehabilitation in improving the functional capacity and strength of frail patients after open-heart surgery.

## 2. Materials and Methods

### 2.1. Study Design, Setting, and Patients’ Selection

This study was a single-center, three-arm, parallel-group, randomized controlled trial. Ethical approval for the study was obtained from the Kaunas Regional Biomedical Research Ethics Committee on 22 July 2021 (No. BE-2-83). On the same day, the first patient was enrolled. It was retrospectively registered in the international database of clinical research studies on 25 April 2024 (ClinicalTrials.gov, registration No. NCT06385041). The retrospective registration occurred because, initially, due to the COVID-19 pandemic and uncertainty about the feasibility of recruiting frail elderly patients after open-heart surgery, the study was initiated as a pilot project. Retrospective registration was performed once the feasibility of completing the study was confirmed. The study was conducted in accordance with the guidelines of the World Medical Association Declaration of Helsinki and followed the Consolidated Standards of Reporting Trials (CONSORT) [26]. The study was carried out in Kulautuva Hospital of Rehabilitation, one of the affiliations of the Hospital of Lithuanian University of Health Sciences, Kauno Klinikos, Lithuania. All patients who arrived in this hospital for medical rehabilitation between July 2021 and May 2024 were invited to participate in this study. Patients were included in the study only when they agreed to participate and signed the informed consent form. The patient was eligible for the study if they met the following criteria: after open-heart surgery (coronary artery bypass grafting, heart valve replacement, or complex surgery), age of more than 65 years, presence of frailty syndrome (score of ≥4 on the Edmonton Frail Scale (EFS)), ability to walk independently (without supportive aids), and ≥150 m based on the 6 min walk test (6MWT). Patients were excluded if they refused to participate in the study, had dysfunction and pathology of the musculoskeletal system, cognitive impairment, severe underlying conditions (mental, visual, and hearing impairments, severe heart insufficiency, severe anemia, postoperative wound healing issues), or other various acute conditions that could limit active participation in the rehabilitation program or assessment.

### 2.2. Patients’ Assessment and Allocation to Groups

A total of 625 patients after open-heart surgery arrived for 20-day cardiac rehabilitation at Kulautuva Hospital of Rehabilitation between July 2021 and May 2024. Of them, 472 did not meet the inclusion criteria; 153 were invited to participate in the study.

On the first rehabilitation day, patients were examined by a physical medicine and rehabilitation physician. Patients were informed about the study verbally and in writing, and medication treatment, management of risk factors, and a protein-enriched diet were prescribed. Based on the medical records of a patient, an individual rehabilitation plan was tailored that included physical therapy, occupational therapy, massage, physical factors, and counseling by a psychologist and a social worker. A unique code for data coding was given to a patient as soon as they received all verbal and written instructions about the study, answered all raised questions, and signed the informed consent form.

**Figure 1:**
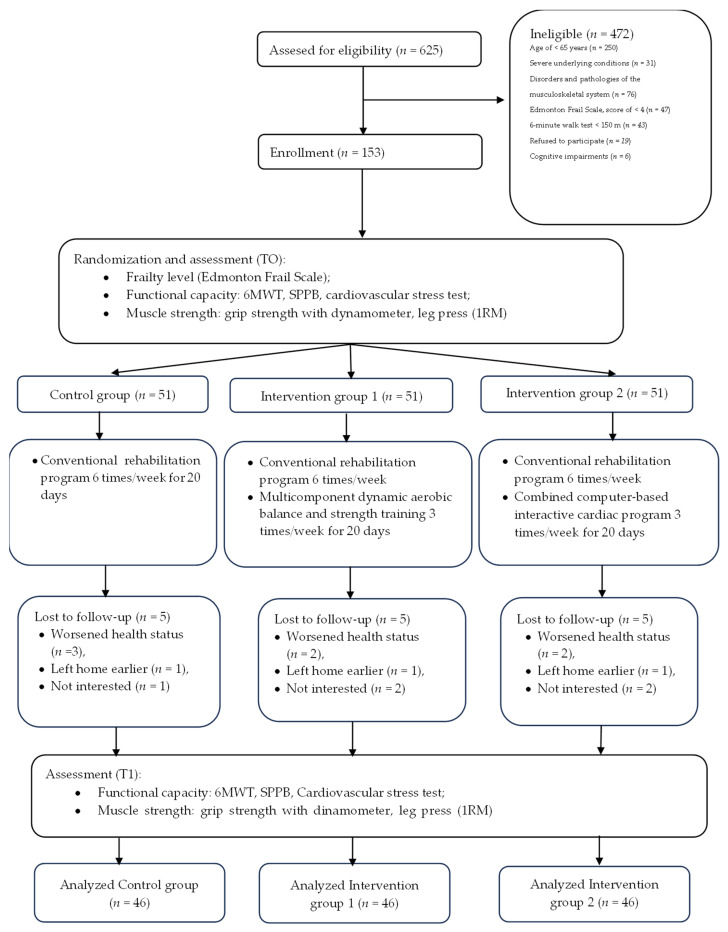
Flowchart of the study.

On the second rehabilitation day, patients were examined to assess their frailty level, physical capacity (6MWT, cycle ergometry, Short Physical Performance Battery (SPPB), and muscle strength (dynamometry and the one-repetition maximum (1RM) test). After examination, patients were randomly allocated to three groups at a ratio of 1:1:1 by using the Research Randomizer program: control group (CG), intervention group 1 (IG-1), and intervention group 2 (IG-2). The flowchart of the study is depicted in Figure 1. Due to the nature of this study, neither the patients nor the investigators’ team could be blinded.

### 2.3. Exercise Interventions

All three groups—CG, IG-1, and IG-2—were given the conventional rehabilitation program. Additionally, patients in the IG-1 group underwent multicomponent dynamic training, and patients in the IG-2 group received the combined computer-based interactive cardiac program (Figure 1). A brief description of all exercise interventions is provided in Table 1, with more detailed explanations presented in a previous publication by our team [27].

All patients who agreed to take part in it and were recruited in this study were examined twice: at the beginning (T0) and end (T1) of rehabilitation. T0 assessment of frailty level, physical capacity (6MWT, cycle ergometry), and muscle strength (dynamometry and lower-limb muscle strength by the 1RM method) was carried out on the second rehabilitation day. During T1 assessment, which took place one day before completing rehabilitation, the same tests (except for the EFS) as during T0 assessment were repeated.

At the beginning of rehabilitation, all patients were examined for their frailty level using the EFS (University of Alberta). The study enrolled only those patients who had an EFS score of ≥4 (from vulnerable to severe frailty).

### 2.4. Study Outcomes

The primary outcome of our study was a change in the SPPB score. The SPPB was used to evaluate the following three components: balance, gait speed, and timed chair stand. Each component is scored from 0 to 4, and the overall SPPB score, ranging from 0 to 12, is obtained by adding the scores of individual components. A score of 0 indicates the worst performance, and a score of 12, the best performance [28].

The secondary outcomes were changes in the 6MWT, peak workload, handgrip strength, and leg press 1RM. Functional capacity was assessed by the 6MWT and cycle ergometry. The 6MWT is a practical and simple clinical test that requires a 30 m hallway. During this test, the distance that a patient can quickly walk on the flat, hard surface in 6 min is measured (the 6MWD). During this test, global and integrated responses of all systems involved in exercise, including pulmonary and cardiovascular systems, systemic and peripheral circulation, neuromuscular system, etc., are assessed. The 6MWT evaluates the submaximal level of functional capacity [29]. Cycle ergometry with a cycle ergometer (Ergoline GmbH, Bitz, Germany) was carried out to determine the patient’s functional capacity and to tailor appropriate and optimal physical workload for them. During this testing carried out by a cardiologist, the electrocardiogram (ECG), heart rate, blood pressure, oxygen saturation, and subjective well-being of a patient are constantly monitored. All parameters are recorded in detail. The initial workload on a cycle ergometer was 25 W, and it was increased gradually every minute by 12.5 W. Workload was increased until one of the following signs appeared: changes in ECG, hypotension (>20 mmHg), hypertension (>230/120 mmHg), chest pain, pronounced shortness of breath, and others. The intensity of the achieved workload was expressed in W and metabolic units (MET) [30].

Handgrip strength was evaluated by using dynamometry, and the muscle strength of the lower extremities was assessed with the 1RM test while performing the leg press. Maximum dynamic leg press strength was assessed using the one-repetition maximum (1RM) test, a reliable and reproducible measure in older adults [31,32,33], with an Ab Hur Oy device (Kokkola, Finland) over a ROM of 90° to ~10° knee flexion. Patients performed 15–25 submaximal repetitions (30–60% estimated maximum) followed by 2 min rest. Testing started at 50–70% estimated maximum, with incremental increases guided by the Borg scale (≤15: +40%; ≥15: +20%) until 10 repetitions could not be completed. Repetitions required proper technique, full ROM, and constant pace; testing was stopped if criteria were not met, pain occurred, or health worsened. The 1RM was indirectly calculated using the Brzycki equation [34,35]. The handgrip strength of both hands was measured with a hydraulic hand dynamometer, Saehan Corporation SH5001 (Changwon, Republic of Korea). The patient was asked to sit in a chair with their shoulder kept in a neutral position and their elbow flexed to 90 degrees. The wrist was fixed in a position of 0 to 30° dorsiflexion. The best result of the three trials was recorded [36].

### 2.5. Adverse Events

Adverse events were defined as any events manifesting with symptoms (e.g., chest pain, arrhythmia, shortness of breath, dizziness, or musculoskeletal injuries) that occurred during the rehabilitation process and were related to physical workload. Patients were constantly monitored by medical staff, and any observed adverse events had to be recorded in the patient’s medical documentation.

### 2.6. Sample Size Calculation

Statistical power calculation (sample size) was performed based on an expected change in the SPPB score. To produce 80% power (β = 0.2) and 95% statistical significance (α = 0.05; *p* = 0.05) with a 1-point difference in the SPPB score, it was estimated that a sample size of 138 patients (46 patients in each of 3 groups) would be sufficient. As the study population comprised older patients who experience arrhythmia, pleural fluid accumulation, wound healing issues, and other complications after open-heart surgery, and their rehabilitation can be discontinued or they are transferred to other hospitals for further inpatient treatment, or they leave rehabilitation due to other personal reasons, there is a possibility that not all patients will complete rehabilitation. Therefore, with a possible attrition rate of 10%, it was calculated that 153 patients had to be enrolled in this study.

### 2.7. Statistical Analysis

Statistical analysis was performed with the Microsoft Excel 2019 and IBM SPSS Statistics 29.0 programs. We applied the Shapiro–Wilk test to check the distribution of continuous data. If continuous data were found to be normally distributed, they were expressed as means with standard deviations (SD), and non-normally distributed, as medians with ranges. Categorical data were expressed as numbers with percentages. Homogeneity of data between the groups of categorical features was checked with the chi-square test and the exact Fisher criterion (in case of a small number of cases). Three independent groups of normally distributed continuous data were compared with ANOVA, and in the case of non-normally distributed data, the nonparametric Kruskal–Wallis test. The multiple comparisons procedure was carried out with the Bonferroni criterion. Dependent groups were compared with the Wilcoxon criterion (non-normally distributed data).

The main analysis was conducted using a per-protocol approach, and only the data of those participants who completed rehabilitation were collected at T0 and T1 assessments and analyzed. As most data in our study did not meet the requirements of parametric testing, we used *z* values to calculate effect size (ES), such as the *r* by using the following formula: *r* = *z*/√N. Effect sizes were interpreted as follows: *r* ≥ 0.50, a large effect; 0.30 ≤ *r* ˂ 0.50, a moderate effect; and 0.10 ≤ *r* ˂ 0.30, a small effect [37]. The level of significance was set at *p* < 0.05.

## 3. Results

A total of 153 patients were enrolled in the study. All three study groups included the same number of patients (*n* = 51); there were more men than women (64.71% vs. 35.29%). Table 2 shows that the comparison of all 3 groups by sex revealed no significant difference (*p* = 0.117). Patients’ height and weight differed significantly between the IG-1 and the IG-2, but there was no significant difference in BMI comparing all three groups (*p* = 0.356). The median age of the study population was 71 years (range, 65–88). The median left ventricular ejection fraction for the whole population was 50% (range, 15–60%). Coronary artery bypass graft surgery was the most frequent surgery performed (64.05%). Among underlying diseases, hypertension (91.5%), dyslipidemia (78.4%), and atrial fibrillation were the most common (30.7%). The median duration from surgery to arrival to medical rehabilitation was 14 days (range, 8–49), and the duration of rehabilitation in the CG, IG-1, and IG-2 was 20 days (range, 9–20), 20 days (range, 9–20), and 19 days (range, 14–20), respectively (*p* = 0.277) (Table 2).

According to the EFS, the median EFS score was 6 (range 4–13), and this corresponds to the mild frailty level. Most patients were vulnerable (41.2%) and mildly frail (42.5%). Assessment of the EFS score showed that all the groups were homogeneous by the frailty level (Table 2).

Of 153 patients enrolled in the study, 15 patients were lost to follow-up due to various reasons (5 patients from each group): 7 patients due to worsened health status, 3 left home earlier, and 5 patients did not show any further interest in continuing rehabilitation (Figure 1). For further analysis, we applied a per-protocol approach, i.e., only the data of those patients who completed rehabilitation were analyzed.

Comparison of patients’ functional capacity and muscle strength before and after rehabilitation revealed statistically significant differences in all parameters of all groups (*p* < 0.05), except for the grip strength of both hands in the IG-1 (Table 3). A comparison of effect sizes before and after rehabilitation revealed a large effect size, ranging from 0.613 to 0.871, for the SPPB score, 6MWD, peak workload, and leg press 1RM. Effect sizes for grip strength of both hands were moderate to large in the CG (0.302 and 0.525, respectively) and in the IG-2 (0.457 and 0.648, respectively); meanwhile, in the IG-1, they were small (0.230 and 0.264, respectively).

Although significant differences in the primary outcome—the SPPB score—were found within each group while performing within-group comparisons (*p* < 0.001), no significant pre-to-post rehabilitation differences were observed comparing all three groups (*p* = 0.939), and the effect sizes were small (Table 4) Meanwhile, changes in some secondary outcomes were positive. The pre- and post-6MWD difference between the control group and the group that received multicomponent training was significant (*p* = 0.014), although the effect size was small (ES = 0.240). Between-group comparisons showed a significant pre- and post-leg press 1RM difference (*p* < 0.001) with a moderate effect size (ES = 0.480) between the control and multicomponent training groups, and with a large effect size (ES = 0.613) between the control and computer-based training groups. No other significant differences were found while performing between-group comparisons, and the effects were very small.

No adverse events related to workload during exercises were recorded in this study.

## 4. Discussion

This study aimed to evaluate and compare the effects of additional exercise interventions, along with conventional cardiac rehabilitation, on the functional capacity and muscle strength of frail patients after open-heart surgery. Studies have demonstrated that additional physical therapy programs are effective in reducing the levels of physical frailty in older people [38]. On the one hand, the findings of our study showed that all applied rehabilitation programs significantly improved the overwhelming majority of functional capacity and muscle strength parameters, but on the other hand, patients who received additional exercise interventions demonstrated greater improvements in functional capacity and muscle strength compared with those who were given conventional postoperative care. These results underscore the potential value of improving conventional rehabilitation protocols in striving to achieve recovery for this vulnerable population. Our study revealed statistically significant changes in the SPPB score—the primary outcome—when performing within-group comparisons. However, there were no significant differences in the SPPB score between the pre- and post-rehabilitation periods among all three groups: conventional, multicomponent, and computer-based. Meanwhile, for 6MWD and leg press 1RM—secondary outcomes—it was shown that additional interventions were more effective in improving these parameters, as pre- and post-differences were significantly greater than conventional rehabilitation.

Literature resources on the effects of interactive training programs, especially for frail patients after open-heart surgery, are scarce [22]; therefore, it was difficult to perform a comparative analysis with other studies in this field, focusing on multicomponent and computer-based exercise programs applied in combination. When discussing the findings of the study, it is important to consider not only statistical significance, but also clinical meaningfulness to more thoroughly assess the clinical importance of the results obtained. A difference of 1 in the SPPB score in our study showed a clinically important change, as based on the literature, clinical importance can be considered when at least a 1-point increase in the cumulative SPPB score is observed [39]. It has been reported that a minimal clinically important difference for change in the 6MWD ranges from 14 to 30.5 m in the population of patients with cardiovascular diseases [40]. In our study, changes were expressed as median differences, and most studies describe the minimal clinically important 6MWD difference as means. Despite this difference in expressing minimal clinically important differences, we found that the median differences in the 6MWD—58.5, 101, and 75 m for the CG, IG-1, and IG-2, respectively—exceeded a previously reported minimal clinically important difference in our study. Therefore, we can state that changes in these parameters in our study were not only statistically but also clinically important.

Literature suggests that a 5-to-6 kg difference in grip strength is clinically important when the patient can feel the change [41]. In our study, grip strength of both hands did not reach this threshold and improved by 0.5 to 2 kg across the groups; therefore, no clinical importance was achieved despite the pre-to-post differences being statistically significant. Information on a clinically important change in the leg press strength is scarce in the literature, but one study found that a 9–10% improvement was clinically meaningful for mobility-limited older adults [42]. For older patients with COPD, a minimal clinically important difference reached 2.5–3 kg [43]. In our study, a pre-to-post rehabilitation difference in leg press 1RM reached 8.1% in the conventional rehabilitation group, and 28.9% as well as 25.6% in the multicomponent and computer-based rehabilitation groups, respectively. Although pre-to-post rehabilitation differences were statistically significant in all groups, we can speculate that only in the groups receiving additional exercise were the differences clinically meaningful. These findings show that strength training is a particularly important aspect for these patients, and by choosing appropriate additional interventions, excellent clinical results can be achieved. Literature search for publications on cycle ergometry data found only one study where a 15.2 W change is mentioned [44]. In our study, we recorded the changes of 11–12.5 W, but we could not draw any conclusions about the clinical importance of our findings, as the above study involved younger patients and a longer study duration.

There is a bidirectional relationship between frailty and cardiovascular disease, and early interventions might be key to reducing the progression of frailty and preventing future cardiovascular events [45]. A major part of our study population comprised vulnerable (42%) and mildly frail (40.6%) patients, and this confirms the predominance of frailty syndrome among patients with heart disease and the need to reduce its prevalence.

A meta-analysis conducted in 2023 evaluated the effects of usual care versus various exercise interventions (balance, endurance, strength, and multicomponent) in adults aged 60 years and older. The results showed no significant differences between exercise types. The most consistent beneficial effects were documented with additional exercises, while performing exercises for 170 min/week [46]. In our study, patients performed additional exercises for 180 min/week, and this confirms that such a duration is sufficient to improve patients’ functional capacity and strength. A randomized controlled trial by Huang et al. [47] showed that after intervention, significant improvements in frailty scores, handgrip strength, and physical activity were documented in the intervention group. In our study, only additional interventions were applied in the intervention groups, and no continuous counseling and support or nutrition assessment were included. This may be the reason why, in the group receiving a multicomponent exercise program, a significant difference was noted only for the 6MWD. Meanwhile, in the study by Huang et al., significantly greater improvements in the frailty score, handgrip strength, and physical activity were recorded for the patients in the intervention group as compared to the control patients. The conclusions of another study carried out by Poli et al. in 2024 are in line with those of our study, that all exercise interventions (multicomponent training, aerobic training, and control groups) are effective, but an additional multicomponent training protocol can improve some outcomes more effectively [48]. Both additional exercise interventions significantly improved patients’ hemodynamic parameters and physical fitness; however, multicomponent training was more effective in improving lower limb strength and dominant hand grip strength [48]. Our study found that the multicomponent exercise program was more effective in improving the 6MWD compared to the control group. Additionally, lower limb strength improved significantly in both exercise groups compared to the control group, with the greatest improvement observed in the computer-based exercise group.

Our results show the beneficial effects of exercise interventions on reducing the development of sarcopenia and its related frailty status. Yang et al. in their meta-analysis concluded that 12-week, in-hospital multicomponent interventions more effectively improved frailty status and enhanced physical functional abilities (muscle strength, gait speed, balance, SPPB, and Timed Up and Go) of older adults aged ≥60 years than interventions conducted out-of-hospital [49]. It is worth noting that, as in our study, cardiac rehabilitation lasted only for 20 days and exercise interventions were performed only in the hospital; we had no possibility of conducting a long-term study. Therefore, long-term outcomes regarding physical performance and strength remain unclear in this context. Undoubtfully, research of longer duration should be conducted in this area as strength, range of motion, and dynamic balance levels can worsen significantly after a 5-month detraining period [50], and continuing home-based training could improve muscle strength, power, and balance [51].

Strength training is a crucial component of cardiac rehabilitation, particularly for frail patients. The meta-analysis by Weng et al. reported that strength training combined with other interventions (multicomponent training) improved frail older adults’ muscle strength, Timed Up and Go, and SPPB, but not gait speed [52]. Another meta-analysis found that both resistance training and multicomponent training could significantly improve muscle strength, while only aerobic exercises included in the conventional rehabilitation program had limited effects. The authors concluded that additional exercises could effectively improve grip strength, knee extension, muscle mass of the lower extremities, walking speed, and functional mobility in older adults suffering from sarcopenia [13].

In our study, multicomponent and computer-based cardiac programs included strength exercises leading to significantly greater pre-to-post rehabilitation differences in muscle strength of the lower extremities compared with the conventional rehabilitation program. Meanwhile, the multicomponent training program was not effective in improving grip strength. Comparison of pre-to-post rehabilitation changes in functional capacity and muscle strength among the groups showed that the computer-based cardiac program was more effective in improving the muscle strength of the lower extremities; meanwhile, multicomponent training was superior in improving functional capacity (6MWD). Another study carried out in 2024 showed that the multicomponent training program reversed frailty status and improved gait speed in pre-frail older adults; however, the efficiency of this multicomponent training protocol was limited in increasing physical activity levels [53]. Previous studies have demonstrated that continuous physical activity and healthy lifestyle behaviors can ensure long-term and effective changes in physical performance [24,53,54]. Another meta-analysis carried out in 2023 reported improvements in muscle strength, gait speed, and aerobic capacity after multicomponent training [55]. The inconsistent findings from these studies indicate that there is no unified opinion, and further detailed research is needed in this field.

All these studies show the benefits of additional (multicomponent) training as compared to cardiac rehabilitation with conventional. Only a few studies have investigated the effects of computer-based training programs. The study by Cacau et al. demonstrated that patients receiving virtual reality interventions after cardiac surgery improved functional performance and reduced pain during the postoperative period [56]. A study similar to ours was conducted by Beigienė et al. (2021), who focused on the evaluation of functional capacity and physical performance of patients after cardiac surgery. Along with conventional cardiac rehabilitation, resistance and balance training, as well as exercises using mechanical devices, were applied, which improved the above outcomes but did not show greater benefits for physical performance results compared with conventional cardiac care [57]. On the contrary, our study found significant results among the groups. The last-mentioned study, as compared to our study, included not only cardiosurgical patients but also patients after percutaneous coronary intervention and had a much smaller sample size. Serdar et al. (in their review) point out that an inadequate sample size can lead to incorrect results in clinical research [58]. Similarly, Khan et al. (in their study) emphasize that subgroups should be prespecified; otherwise, results can be misinterpreted [59]. It is especially relevant in studies involving cardiac patients, where treatment effects can differ among patient groups. Therefore, our study enrolled a more specific population of cardiac patients—namely, frail patients after open-heart surgery—and the sample size was increased accordingly.

Summarizing, the obtained results allow for the assumption that multicomponent intervention programs are more suitable for the improvement of endurance (6MWD), while computer-based programs are more suitable for the improvement of muscle strength (leg press). The implementation of these additional exercises into clinical practice raises some feasibility and cost-effectiveness questions: the multicomponent program does not require specialized equipment and is more cost-effective, but it could present a challenge due to the complexity related to its organization (dosing, monitoring, etc.). The integration of computer-based rehabilitation in clinical practice would offer some advantages, including better accessibility, the possibility of involving a greater number of patients in rehabilitation, and the provision of personalized and accurate recommendations for continuing physical exercise at home. Moreover, such programs could reduce the need for personnel in clinical settings and overall health care costs compared to conventional interventions provided by health care specialists. However, the practical implementation of computer-based programs could be limited by patients’ digital literacy, access to technologies, and the costs of initial equipment integration. Further research is warranted to evaluate cost-effectiveness and practical implementation strategies striving to optimize the integration of computer-based programs in daily clinical practice.

This study has several limitations. First, the study was conducted in a single Lithuanian health care facility. Second, the duration of rehabilitation was short (up to 20 calendar days), and no post-discharge follow-up was conducted. With such short rehabilitation, no functional gains could be sustained. Without follow-up, no long-term effects on the parameters of physical performance and muscle strength could be evaluated. Third, the study population mainly comprised vulnerable and mildly frail patients; therefore, it raises the question of whether the results of this study can be generalized to the population of patients with higher levels of frailty. The inclusion criteria, such as a walking distance of ≥150 m and open-heart surgery, could limit the number of other patients who could have been included in the study (after percutaneous coronary intervention, walking distance of <150 m, etc.).

## 5. Conclusions

Within-group comparison showed that all three rehabilitation programs are effective in improving almost all parameters of physical performance and muscle strength in frail patients after open-heart surgery. However, between-group comparisons indicated that computer-based interventions were more effective in improving leg press 1RM with a large effect size, while multicomponent training resulted in more effective gains in the 6MWD, although with a small effect size. These findings suggest that in clinical practice, computer-based exercise programs may be more suitable for patients with muscle weakness, while multicomponent exercise programs, for those with reduced endurance.

## Figures and Tables

**Table 1:** Brief description of exercise interventions given to the study groups.

Type of Activity	Description
(CG) Conventional Rehabilitation Program	
Aerobic training	Frequency: 6 times/week Intensity: light-moderate (Borg 9–13); intensity 37–45% VO_2max_, peak heart rate (HR_peak_) 57–63%, heart rate reserve from 30–39% Duration: up to 40–45 min Type: aerobic endurance with cycle ergometer
Stretching exercises	Frequency: 7 times/week Intensity: light-to-moderate (Borg 9–13) Duration: 10 min Type: passive stretching and ROM for major muscle groups
Breathing exercises	Frequency: 7 times/week Intensity: light (Borg 9–11) Duration: 10 min Type: diaphragmatic breathing
(IG-1) Conventional rehabilitation program plus multicomponent dynamic training	
Aerobic activity	Frequency: 3 times/week Intensity: moderate (Borg 12–13) Duration: 20–30 min Type: aerobic endurance (stair climbing, walking, cycling or other cyclic aerobic activity)
Sensorimotor training	Frequency: 3 times/week Intensity: light-to-moderate (Borg 9–13) Duration: 15–20 min Type: sensorimotor, balance, coordination with balance platforms
Muscle strength	Frequency: 3 times/week Intensity: starting from light (30% of 1RM) and increasing to moderate-vigorous (Borg 9–17) Extent: 8–10 exercises (3 sessions, 10 repetitions) Type: strength training with resistance bands and free weights.
Flexibility	Frequency: 3 times/week Intensity: light (Borg 9–11) Duration: 5–10 min Type: ROM, stretching
(IG-2) Conventional rehabilitation program plus combined computer-based interactive cardiac program	
Gait improvement and aerobic activity	Frequency: 3 times/week Intensity: moderate (Borg 12–13) Duration: 20–30 min Type: gait training and aerobic endurance training with Biodex GaitTrainer™^3^ treadmill and Zebris FDMT
Sensorimotor training	Frequency: 3 times/week Intensity: light-to-moderate (Borg 9–13) Duration: ~20 min, each training mode lasted for 3 min Type: sensorimotor, balance, coordination with Biodex Balance SD
Muscle strength	Frequency: 3 times/week Intensity: starting from light (30% of 1RM) and increasing to moderate-vigorous (Borg 9–17) Extent: 8–10 exercises (3 sessions, 10 repetitions) Type: strength with HUR device

**Table 2:** General characteristics of all patients enrolled in the study by the groups.

Characteristic	CG (*n* = 51)	IG-1 (*n* = 51)	IG-2 (*n* = 51)	*p* *	*p* ** CG vs. IG-2	*p* ** IG-1 vs. IG-2	*p* ** CG vs. IG-1
Age, years, median (range)	73 (65; 88)	71 (65; 87)	71 (65; 87)	0.314	0.435	>0.99	0.801
Sex, n (%)							
Male	38 (74.5)	28 (54.9)	33 (64.7)	0.177	NA		
Female	13 (25.5)	23 (45.1)	18 (35.3)				
Height, m, median (range)	1.70 (1.52; 1.85)	1.66 (1.49; 1.78)	1.71 (1.52; 1.85)	**0.006**	>0.99	**0.006**	0.058
Weight, kg, median (range)	77 (52; 118)	76 (46; 107)	80 (59; 111)	**0.025**	0.519	**0.020**	0.529
BMI, kg/m^2^, median (range)	27.70 (20.31; 37.88)	27.44 (18.71; 41.02)	28.41 (20.42; 37.34)	0.356	>0.99	0.457	> 0.99
LVEF, %	50 (30; 55)	50 (15; 58)	50 (34; 60)	0.186	0.229	0.581	> 0.99
Beginning of CR after surgery, days	15 (9; 45)	14 (10; 49)	14 (10;49)	0.060	0.062	0.324	> 0.99
CR duration, days	20 (9; 20)	20 (9; 20)	19 (14; 20)	0.227	>0.99	0.257	> 0.99
Surgery type, n (%)							
CABG	29 (56.9)	32 (62.7)	37 (72.5)	0.573	NA		
Heart valve surgery	9 (17.6)	7 (13.7)	6 (11.8)				
AVR-CABG	13 (25.5)	12 (23.5)	8 (15.7)				
Current smokers, n (%)	2 (3.9)	7 (13.7)	5 (39.8)	0.225		NA	
Comorbidities, n (%)							
Diabetes	8 (15.7)	7 (13.7)	11 (21.6)	0.548		NA	
COPD	0 (0.0)	2 (3.9)	1 (2.0)	0.361		NA	
Atrial fibrillation	18 (35.3)	14 (27.5)	15 (29.4)	0.671		NA	
Arterial hypertension	47 (92.2)	47 (92.2)	46 (90.2)	0.919		NA	
Depression	1 (2.0)	1 (2.0)	0 (2.0)	0.603		NA	
Diseases of the musculoskeletal system	5 (9.8)	2 (3.9)	9 (17.6)	0.076		NA	
Oncological diseases	6 (11.8)	5 (9.8)	3 (5.9)	0.577		NA	
Dyslipidemia	42 (82.4)	41 (80.4)	37 (72.5)	0.444		NA	
Total EFS, score, median (range)	6 (4; 10)	6 (4; 13)	6 (4; 11)	0.748	>0.99	>0.99	>0.99
Frailty classification, n (%)							
Vulnerable	21 (41.2)	23 (45.1)	19 (37.3)	0.800	NA		
Mild Frailty	22 (43.1)	21 (41.2)	22 (43.1)				
Moderate Frailty	7 (13.7)	6 (11.8)	9 (17.6)				
Severe Frailty	1 (2.0)	1 (2.0)	1 (2.0)				

BMI, body mass index; CR, cardiac rehabilitation; LVEF, left ventricular ejection fraction; AVR-CABG, aortic valve replacement in combination with coronary artery bypass grafting; COPD, chronic obstructive pulmonary disease; EFS, Edmonton Frail Scale; NA, not applicable. * Categorical data were compared with the chi-square test; independent nonnormally distributed data, with the Kruskal–Wallis test. ** *p*-values for multiple group comparisons procedure obtained by the Bonferroni correction. Values in Bold indicate statistical significance.

**Table 3:** Effectiveness of cardiac rehabilitation on functional capacity and muscle strength by the groups.

Parameters	CG (*n* = 46)		ES	*p* *	IG-1 (*n* = 46)		ES	*p* *	IG-2 (*n* = 46)		ES	*p* *
	T0	T1			T0	T1			T0	T1		
SPPB, score	10 (5; 12)	12 (7; 12)	0.613	**<0.001**	10 (6; 12)	11 (6; 12)	0.744	**<0.001**	10 (6; 12)	11 (8; 12)	0.664	**<0.001**
6MWD, m	283.5 (151; 545)	358 (160; 570)	0.813	**<0.001**	276 (150; 418)	398.5 (269; 736)	0.871	**<0.001**	300 (149; 456)	392.5 (228; 533)	0.808	**<0.001**
Peak workload, W	42.5 (28; 82)	55 (36; 109)	0.831	**<0.001**	48.5 (27; 91)	60 (29; 136)	0.852	**<0.001**	50 (34; 90)	61 (36; 110)	0.725	**<0.001**
Grip strength, kg												
Right, kg	32 (16; 64)	33 (16; 56)	0.302	**<0.001**	27 (12; 45)	28 (14; 45)	0.264	0.123	34 (12; 50)	34 (15; 52)	0.457	**0.001**
Left, kg	31 (10; 54)	32 (12; 56)	0.525	**<0.001**	24 (12; 45)	25 (10; 47)	0.230	0.262	27 (10; 48)	29 (9; 52)	0.648	**<0.001**
Leg press 1RM, kg	30 (16; 47)	33 (17; 47)	0.629	**<0.001**	32 (17; 62)	45 (19; 65)	0.754	**<0.001**	34.5 (16; 56)	47 (23; 65)	0.820	**<0.001**

Values are expressed as median (range). 6MWD, 6 min walk distance; SPPB, Short Physical Performance Battery; RM, repetition maximum; ES, effect size. * *p* value by the Wilcoxon test. Values in Bold indicate statistical significance.

**Table 4:** Pre- and post-rehabilitation differences in the parameters of functional capacity and muscle strength between the groups.

Parameters	T1–T0			*p* *	CG vs. IG-2		IG-1 vs. IG-2		CG vs. IG-1	
	CG (*n* = 46)	IG-1 (*n* = 46)	IG-2 (*n* = 46)		ES	*p* **	ES	*p* **	ES	*p* **
SPPB, score	1 (–2; 6)	1 (–1; 4)	1 (–1; 5)	0.939	0.013	0.913	0.034	0.729	0.027	0.812
6MWD, m	58.5 (–113; 361)	101 (11; 318)	75 (–124; 221)	**0.047**	0.110	0.359	0.174	0.126	0.240	**0.014**
Peak workload, W	11 (–8; 37)	13 (–4; 45)	11.5 (–18; 33)	0.572	0.078	0.438	0.108	0.313	0.021	0.816
Grip strength, kg										
Right hand	1 (–12; 10)	2 (–6; 7)	2 (–6; 10)	0.690	0.086	0.414	0.067	0.517	0.017	0.865
Left hand	2 (–6; 7)	0.5 (–7; 11)	2 (–6; 9)	0.262	0.038	0.737	0.159	0.120	0.129	0.222
Leg press 1RM, kg	3 (–7; 12)	9 (–15; 34)	11.5 (–11; 33)	**<0.001**	0.613	**<0.001**	0.127	0.215	0.480	**<0.001**

Values are expressed as median (range). 6MWD, 6 min walk distance; SPPB, Short Physical Performance Battery; 1RM, 1 repetition maximum; ES, effect size. * *p* value by Kruskal–Wallis test; ** *p* values for multiple comparisons procedure obtained by the Bonferroni correction. Values in Bold indicate statistical significance.

## Data Availability

The data presented in this study are available on request from the corresponding author. The data is not publicly available due to ethical restrictions and data protection policies.
